# Supplementary material for: Using ephaptic coupling to estimate the synaptic cleft resistivity of the calyx of Held synapse
Source: PLoS Comput Biol. 2021 Oct 26;17(10):e1009527. doi: 10.1371/journal.pcbi.1009527 (PMC8570497; doi:10.1371/journal.pcbi.1009527)
Supplement: S1 Appendix — In this Appendix we provide, given the electronic model, the analytical relations for the cleft potential, the VC prespike and the CC prespike, and we address how the presynaptic calcium current couples to the postsynaptic recording. (PDF) [file pcbi.1009527.s001.pdf]

S1. Appendix A1. Analysis of the electronic model & A2. the calcium prespike

A1. Analysis of the electronic model

Following Kirchhoff's current law, the sum of the currents that enter the cleft must be equal to the sum of the currents leaving the cleft:

$i_{leave} = i_{enter}$ $(A1.1)$.

The currents that enter the cleft are either resistive or capacitive, coming either from the presynaptic or the postsynaptic side; we also included the conductances of presynaptic voltage-gated channels (g_{ion}) (Fig 2B):

$$i_{enter} = g_{pre}\left(v_{pre} - v_{pre,rest}\right) + c_{pre}\frac{dv_{pre}}{dt} + g_{syn}\left(v_{post} - v_{post,rest}\right) + c_{syn}\frac{dv_{post}}{dt} + \sum g_{ion}(v_{pre} - v_{ion}) \qquad (A1.2)$$

where v_{pre} and v_{post} are the pre- and the postsynaptic membrane potential relative to the interstitial fluid, respectively, $v_{pre,rest}$ and $v_{post,rest}$ are the pre- and the postsynaptic resting membrane potential relative to the interstitial fluid, respectively, c_{pre} and c_{syn} are the capacitances of the pre- and the postsynaptic membrane facing the cleft, respectively, and g_{pre} and g_{syn} are the resting, leak conductance of the pre- and postsynaptic membrane facing the cleft, respectively (Fig 2). The summation sign indicates the summation of the different types of voltage-gated channels, each with their own time- and voltage-dependent conductance g_{ion} and their own reversal potential v_{ion} relative to the extracellular fluid.

The currents leaving the cleft are resistive through a cleft leak conductance (g_{cl}), and resistive and capacitive through the presynaptic and postsynaptic side:

$$i_{leave} = v_{cl}\left(g_{cl} + g_{pre} + g_{syn} + \sum g_{ion}\right) + \left(c_{pre} + c_{syn}\right)\frac{dv_{cl}}{dt} \qquad (A1.3)$$

This gives the full equation for the cleft (combine Eqs. S1.1-3), similar to ref. [1]:

$$v_{cl}\left(g_{cl} + g_{syn} + g_{pre} + \sum g_{ion}\right) + \left(c_{pre} + c_{syn}\right)\frac{dv_{cl}}{dt} = g_{pre}\left(v_{pre} - v_{pre,rest}\right) + c_{pre}\frac{dv_{pre}}{dt} + g_{syn}\left(v_{post} - v_{post,rest}\right) + c_{syn}\frac{dv_{post}}{dt} + \sum g_{ion}(v_{pre} - v_{ion}) \qquad (A1.4)$$

We simplify the full equation by assuming that the postsynaptic currents entering the cleft (g_{syn} (v_{post} - $v_{post,rest}$) + $c_{syn}dv_{post}/dt$) are much smaller than the presynaptic currents entering the cleft, as is the case when the prespike is very small, and therefore can be neglected. For the calyx of Held synapse, the

postsynaptic, cleft-facing, resting conductance is indeed rather small (g_{syn} < 3 nS, excluding the conductance of the EPSC), and the prespike is at most a few mV. The presynaptic capacitive currents during the presynaptic AP are much larger than the resistive currents, as g_{pre} < 5 nS, generating currents less than 0.6 nA (<120 mV x 5 nS), while the presynaptic capacitive current can easily be 5 nA (500 V/s x 10 pF). We thus derive the following, simplified equation:

$$v_{cl}\left(g_{cl}+\sum g_{ion}\right)+\left(c_{pre}+c_{syn}\right)\frac{dv_{cl}}{dt}=c_{pre}\frac{dv_{pre}}{dt}+\sum g_{ion}\left(v_{pre}-v_{ion}\right) \qquad (A1.5)$$

For a given synapse, we can distinguish two extreme scenarios depending on the route currents entering the cleft may take: resistive versus capacitive dissipation. If the cleft currents mainly dissipate via capacitive routes (membrane, $v_{cl}(g_{cl} + \Sigma g_{ion})<<(c_{pre} + c_{syn})dv_{cl}/dt$). Eq. A1.5 can be simplified to:

$$\left(c_{pre}+c_{syn}\right)\frac{dv_{cl}}{dt}=c_{pre}\frac{dv_{pre}}{dt}+\sum g_{ion}\left(v_{pre}-v_{ion}\right) \qquad (A1.6)$$

$$\frac{dv_{cl}}{dt}=\frac{c_{pre}}{c_{pre}+c_{syn}}\frac{dv_{pre}}{dt}+\frac{1}{c_{pre}+c_{syn}}\sum g_{ion}\left(v_{pre}-v_{ion}\right) \qquad (A1.7)$$

$$v_{cl}=\int\left(\frac{c_{pre}}{c_{pre}+c_{syn}}\frac{dv_{pre}}{dt}+\frac{1}{c_{pre}+c_{syn}}\sum g_{ion}\left(v_{pre}-v_{ion}\right)\right)dt$$
$$=\frac{c_{pre}}{c_{pre}+c_{syn}}v_{pre}+\frac{1}{c_{pre}+c_{syn}}\int\left(\sum g_{ion}\left(v_{pre}-v_{ion}\right)\right)dt$$
$$+v_{constant} \qquad (A1.8)$$

The VC prespike becomes (using eq. A1.7):

$$-i_{rec}=c_{syn}\frac{dv_{cl}}{dt}=\frac{c_{syn}}{c_{pre}+c_{syn}}\left(c_{pre}\frac{dv_{pre}}{dt}+\sum g_{ion}\left(v_{pre}-v_{ion}\right)\right) \qquad (A1.9)$$

Note that in the capacitive dissipation scenario, cleft currents dissipate as a postsynaptic as well as a presynaptic capacitive current. The relative fraction that dissipates through the presynaptic and postsynaptic membrane equals $c_{pre}\,(c_{pre}+c_{syn})^{-1}$ and $c_{syn}\,(c_{pre}+c_{syn})^{-1}$, respectively. In other words, the presynaptic currents are partially reflected back to the presynaptic side in this scenario. For the calyx of Held synapse, where the presynaptic and postsynaptic capacitance are nearly equal, the relative fraction equals 0.5.

In the resistive dissipation scenario we consider the case where the cleft currents mainly dissipate via resistive routes ($v_{cl}(g_{cl} + \Sigma g_{ion})>>(c_{pre} + c_{syn})dv_{cl}/dt$).We can neglect the contribution of the capacitive

currents induced by the cleft potential to derive the equation for the cleft potential. Eq. A1.5 can then be simplified to:

$$v_{cl}\left(g_{cl}+\sum g_{ion}\right)=c_{pre}\frac{dv_{pre}}{dt}+\sum g_{ion}\left(v_{pre}-v_{ion}\right) \qquad (A1.10)$$

$$v_{cl}=\left(g_{cl}+\sum g_{ion}\right)^{-1}\left(c_{pre}\frac{dv_{pre}}{dt}+\sum g_{ion}\left(v_{pre}-v_{ion}\right)\right) \qquad (A1.11)$$

An additional simplification is justified if $g_{cl} >> \Sigma g_{ion}$:

$$v_{cl}=\left(g_{cl}\right)^{-1}\left(c_{pre}\frac{dv_{pre}}{dt}+\sum g_{ion}\left(v_{pre}-v_{ion}\right)\right) \qquad (A1.12)$$

In this case, v_{cl} becomes a scaled sum of the first derivative of the presynaptic AP and ionic currents through voltage-gated channels.

The postsynaptic prespike, when recorded in voltage-clamp, still equals the capacitive current over the postsynaptic membrane, which now is assumed to be much smaller than the currents that are present in the cleft:

$$-i_{rec} = c_{syn}\frac{dv_{cl}}{dt}=c_{syn}\frac{d}{dt}\left(\left(g_{cl}\right)^{-1}\left(c_{pre}\frac{dv_{pre}}{dt}+\sum g_{ion}\left(v_{pre}-v_{ion}\right)\right)\right)$$

$$=\frac{c_{syn}c_{pre}}{g_{cl}}\frac{d^2v_{pre}}{dt^2}+\frac{c_{syn}}{g_{cl}}\sum\frac{d}{dt}\left(g_{ion}\left(v_{pre}-v_{ion}\right)\right) \qquad (A1.13)$$

For the CC prespike, in both situations, the current entering the postsynaptic cell via the cleft membrane must be equal to the current leaving the cell:

$$c_{post}\frac{dv_{post}}{dt}+g_{post}\left(v_{post}-v_{post,rest}\right)=-i_{rec}=c_{syn}\frac{dv_{cl}}{dt}+g_{syn}v_{cl} \qquad (A1.14)$$

Where c_{post} and g_{post} are the total postsynaptic capacitance and conductance, respectively (Fig 2). The shape of the CC prespike will depend on the cleft potential, the cleft-facing capacitance and the conductance of the cleft-facing membrane, allowing a large range of different prespike shapes (reflecting the integral of the cleft potential, the cleft potential itself, or the first derivative of the cleft potential).

We can simplify the notation of some equations by defining a cleft time constant ($\tau_{cl} = c_{cl}/g_{cl}$), and $c_{cl} = c_{syn} = c_{pre}$. The equations then become remarkably simple:

$$v_{cl} = \frac{1}{g_{cl}}\left(c_{cl}\frac{dv_{pre}}{dt} + \sum g_{ion}\left(v_{pre} - v_{ion}\right)\right) = \frac{i_{cleft}}{g_{cl}} \qquad (A1.15)$$

$$-i_{rec} = c_{cl}\frac{dv_{cl}}{dt} = \tau_{cl}\frac{di_{cleft}}{dt} \qquad (A1.16)$$

$$c_{post}\frac{dv_{post}}{dt} + g_{post}v_{post} = -i_{rec} = \tau_{cl}\frac{di_{cleft}}{dt} = c_{cl}\frac{dv_{cl}}{dt} \qquad (A1.17)$$

If we furthermore assume that $g_{post}\,(v_{post} - v_{post,rest}) << c_{post}\,dv_{post}/dt$:

$$c_{post}\frac{dv_{post}}{dt} = \tau_{cl}\frac{di_{enter}}{dt} = c_{cl}\frac{dv_{cl}}{dt}$$

$$v_{post} = \frac{c_{cl}}{c_{post}g_{cl}}i_{enter} + v_{post,rest} = \frac{c_{cl}}{c_{post}}v_{cl} + v_{post,rest} \qquad (A1.18)$$

The cleft potential is then defined by Ohm's law: the ratio of the net current that runs through the cleft and the cleft leak conductance. The VC prespike is defined by the first derivative of the net currents scaled by the cleft time constant. The CC prespike is defined by the cleft potential and the fraction of the postsynaptic capacitance facing the cleft.

A2. The calcium prespike

This Appendix deals with the question how the recorded calcium current contributes to the VC prespike. The current i_{ion} that runs through the presynaptic cleft-facing membrane can leak away either capacitively or resistively similar to the capacitive currents from the presynaptic AP. The recorded calcium current typically closely follows a Gaussian function [2,3]:

$$i_{ca} = i_{max}e^{t^2/(-2\tau_{Ca}{}^2)} \qquad (A2.1)$$

For the capacitive dissipation scenario, we rewrite A1.9:

$$-i_{rec} = c_{syn}\frac{dv_{cl}}{dt} = c_{pre}\frac{d\left(v_{pre} - v_{cl}\right)}{dt} + i_{Ca} \qquad (A2.2)$$

When we subtract the passive prespike ($i_{passive\text{-}cap}$) from the prespike recording, we arrive at the calcium prespike:

$$-i_{rec} + i_{passive-cap} = i_{ca} \qquad (A2.3)$$

For the resistive dissipation scenario, we rewrite A1.16 and we again subtract the passive prespike ($i_{passive\text{-}res}$) to arrive at the calcium prespike:

$$-i_{rec} + i_{passive-res} = \tau_{cl}\frac{di_{ca}}{dt} = \tau_{cl} i_{max}\frac{d}{dt}\left(e^{t^2/(-2\tau_{Ca}{}^2)}\right) = \frac{\tau_{cl} i_{max}}{\tau_{Ca}{}^2}\, t\, e^{t^2/(-2\tau_{Ca}{}^2)} \qquad (A2.4)$$

This equation shows two peaks at $t = \pm\tau_{Ca}$ with an amplitude of

$$-i_{rec}(\tau_{Ca}) + i_{passive-res}(\tau_{Ca}) = \pm\frac{\tau_{cl} i_{max}}{\tau_{Ca}{}^2}\,\tau_{Ca}\, e^{(\pm\tau_{Ca})^2/(-2\tau_{Ca}{}^2)} = \pm\frac{\tau_{cl}}{\tau_{Ca}\sqrt{e}} i_{max} \qquad (A2.5)$$

We thus define a simple scaling factor $\tau_{cl}\,(\tau_{Ca}\sqrt{e})^{-1}$ that relates the amplitude of the presynaptic calcium current to the amplitude of the calcium prespike.

References

1. Savtchenko LP. Bilateral processing in chemical synapses with electrical 'ephaptic' feedback: a theoretical model. Math Biosci. 2007;207(1):113-37. doi: 10.1016/j.mbs.2006.09.016. PubMed PMID: 17112549.
2. Borst JGG, Helmchen F. Calcium influx during an action potential. In: Conn PM, editor. Methods in Enzymology. Ion Channels. 293: Academic Press; 1998. p. 352-71.
3. Borst JGG, Sakmann B. Calcium current during a single action potential in a large presynaptic terminal of the rat brainstem. J Physiol. 1998;506:143-57. doi: 10.1111/j.1469-7793.1998.143bx.x.
