# Supplementary material for: Using ephaptic coupling to estimate the synaptic cleft resistivity of the calyx of Held synapse
Source: PLoS Comput Biol. 2021 Oct 26;17(10):e1009527. doi: 10.1371/journal.pcbi.1009527 (PMC8570497; doi:10.1371/journal.pcbi.1009527)
Supplement: S2 Appendix — In this appendix we provide the geometrical relation for the cleft voltage profile for a radially-symmetric synapse as a result of the capacitive currents from the presynaptic action potential, the postsynaptic current this will generate, how the cleft conductance relates to the extracellular resistivity assuming a radially-symmetric synapse, what these relations become for a finger-like synapse, and how the transformation from a radially-symmetric synapse to synapse composed of multiple fingers changes the cleft potential profile. (PDF) [file pcbi.1009527.s002.pdf]

S2 Appendix. B. Geometry

Question B1: What is the voltage profile in the synaptic cleft for a simplified synapse?

We assume a radially-symmetrical synapse with radius r and cleft height h. The capacitive current that enters the synaptic cleft equals:

$$i_{cap} = c_m v'_{pre} = C_m \pi r^2 v'_{pre} \qquad \text{(B1.1)}$$

with v'_{pre} indicating the first temporal derivative of the presynaptic AP. We assume that the current is homogeneously distributed over the cleft-facing membrane and that all currents within the cleft run radially towards the interstitial fluid (resistive dissipation). The current density $J(x)$ can be defined as a quasi-static function of the radial location x, where 0 indicates the center and r the synapse edge. J(x) will be equal to the current that runs through the cleft-facing membrane from the center to radius x divided by the surface through which it escapes, $A_{esc}(x)$. This surface is the surface of an open cylinder with radius x and height h:

$$J(x) = \frac{i(x)}{A_{esc}(x)} = \frac{\pi x^2 C_m v'_{pre}}{2\pi x h} = \frac{C_m v'_{pre}}{2h} x \qquad \text{(B1.2)}$$

Based on $J(x)$, the electric field can be defined using a homogeneous extracellular cleft resistivity R_{ex}, where the negative sign reflects the convention that current runs down the voltage gradient:

$$-\frac{dv(x)}{dx} = R_{ex} J(x) = \frac{R_{ex} C_m v'_{pre}}{2h} x \qquad \text{(B1.3)}$$

The voltage profile relative to the cleft edge is the integral of equation B1.3 from x to r:

$$v(x) = \int_x^r \left(-\frac{R_{ex} C_m v'_{pre}}{2h} x\right) dx = -\frac{R_{ex} C_m v'_{pre}}{2h} \int_x^r (x) dx = \frac{R_{ex} C_m v'_{pre}}{4h} (r^2 - x^2) \qquad \text{(B1.4)}$$

We can also rewrite equation B1.4 as a relative distance to the cleft edge:

$$v(x/r) = \frac{r^2 R_{ex} C_m v'_{pre}}{4h} (1 - (x/r)^2) \qquad \text{(B1.5)}$$

The voltage profile will apply as long as all current runs radially to the extracellular matrix. When this is not the case -for instance, when currents enter into the postsynaptic cell or return through the presynaptic cleft-facing membrane- the current density will be reduced, and the voltage gradient will be reduced as well.

Question B2: How much current will be generated at the postsynaptic membrane?

We assume that the currents that run through the postsynaptic membrane are purely capacitive:

$$i_{post} = \int_{x=0}^{r} i_{cap}(x)dx = \int_{x=0}^{r} (C_m A(x) v'(x))dx \qquad \text{(B2.1)}$$

Here, the current through an annulus of membrane (at x, with thickness dx) will depend on the radial location as the surface of the annulus equals $2\pi x dx$ and the cleft potential is also defined by the radial location (equation B1.4). Only v'_{pre} will be affected by the time derivative, which will become the second time derivative (v''_{pre}) . The equation then becomes:

$$i_{post} = C_m \int_{x=0}^{r} \left(2\pi x \ \frac{R_{ex} C_m v''_{pre}}{4h} \ (r^2 - x^2) \right) dx$$

$$= \frac{\pi R_{ex} C_m^2 v''_{pre}}{2h} \int_{x=0}^{r} (x \ (r^2 - x^2))dx$$

$$= \frac{\pi R_{ex} C_m^2 v''_{pre}}{2h} \ \left[\frac{1}{2} r^2 x^2 - \frac{1}{4} x^4 \right]_{x=0}^{r}$$

$$= \frac{\pi R_{ex} C_m^2 v''_{pre}}{8h} r^4 \qquad \text{(B2.2)}$$

Question B3: How does the cleft resistivity relate to the cleft conductance?

We noted that B2.2 resembles A1.13 without the voltage-gated currents:

$$i_{post} = \frac{{c_{cl}}^2}{g_{cl}} v''_{pre} = \frac{(C_m \pi r^2)^2}{g_{cl}} v''_{pre} \qquad \text{(B3.1)}$$

By combining equation B2.2 and B3.1 we find that g_{cl} relates to the cleft resistivity:

$$g_{cl} = \frac{8\pi h}{R_{ex}} \qquad \text{(B3.2)}$$

Combining equations B3.1 and B3.2 yields an alternative way to calculate the extracellular resistivity:

$$R_{ex} = \frac{i_{post}\, 8\pi h}{c_{cl}^2\, v''_{pre}} \qquad \text{(B3.3)}$$

This equation holds as long as the cleft currents dissipate resistively and the contribution of voltage-gated currents are negligible.

Question B4. What are the relations for a finger-like synapse?

We assume a sheet-like synapse with a cleft height *h*, a constant width of 2*r*, and a length *L* that is closed at the longitudinal ends. Any current that enters the synaptic cleft cannot escape along the longitudinal axis, leaving only the transversal route to the interstitial space. Examples of such synapses are finger-like synapses, such as the neuromuscular junction or the endbulb of Held synapse for which we ignore the longitudinal endings, or deeply-invaginating, cylindrical synapses (spinules) with a circular longitudinal axis (*i.e.* a rolled-up sheet forming a cylinder). Because of the constant width, the voltage profile will be identical along the longitudinal axis, varying only along the synapse width. The capacitive current will be equal to $i_{cap}(x) = C_m v'_{pre} drdl$ (B4.1).

The current density *J* at each location will be:

$$J(x) = \frac{i(x)}{A_{esc}(x)} = \frac{C_m v'_{pre} x dl}{h dl} = \frac{C_m v'_{pre}}{h} x \qquad \text{(B4.2)}$$

The voltage gradient then is:

$$-\frac{dv(x)}{dx} = R_{ex} J(x) = \frac{R_{ex} C_m v'_{pre}}{h} x \qquad \text{(B4.3)}$$

And the voltage at x is:

$$v(x) = -\frac{R_{ex} C_m v'_{pre}}{h} \int_x^r (x) dx = \frac{R_{ex} C_m v'_{pre}}{2h} (r^2 - x^2) \qquad \text{(B4.4)}$$

The changes in the cleft potential will generate a postsynaptic, capacitive current. To calculate the total current from the finger-like synapse, we sum the current along the two dimensions:

$$i_{fin} = \int_{l=0}^{L} 2\int_{x=0}^{r} i_{cap}(x)dx\,dl = \int_{l=0}^{L} 2\int_{x=0}^{r} C_m A v'(x)dx\,dl$$

$$= \int_{l=0}^{L} 2\int_{x=0}^{r} C_m \frac{R_{ex}C_m v''_{pre}}{2h}(r^2 - x^2)dx\,dl$$

$$= \frac{R_{ex}C_m^2 v''_{pre}}{h}\int_{l=0}^{L}\int_{x=0}^{r}(r^2 - x^2)dx\,dl = \frac{R_{ex}C_m^2 v''_{pre}}{h}\int_{l=0}^{L}\left[xr^2 - \frac{1}{3}x^3\right]_{x=0}^{r} dl$$

$$= \frac{2R_{ex}C_m^2 v''_{pre} r^3}{3h}\int_{l=0}^{L} dl = \frac{2R_{ex}C_m^2 v''_{pre} r^3 L}{3h} \qquad \text{(B4.5)}$$

For the sheet-like synapse, x=0 indicates the cleft center and currents can run in two, transversal directions. We therefore multiply the capacitive current by 2 at the start of the derivation of B4.5. For a spinule synapse, no current can escape from the ‘deep’ end of the cylinder (at x=0) and therefore the currents run in a single, transversal direction. The right-hand side of B4.5 should therefore be halved for spinule-like synapses or other deeply-invaginating synapses, neglecting any contribution from the cup-shaped cleft at the deep end. The characteristic difference between the radial-like or sheet-like synapse is whether the escape area along the longitudinal axis increases or stays constant, respectively. Following the logic outlined in B1, B2 and B4, one can calculate the cleft potentials and postsynaptic currents for other synaptic geometries.

Question B5. How does fenestration change the cleft potential and the VC prespike?

To assess the impact of fenestration on the cleft potential and the VC prespike, we compare a radially-symmetric synapse and a synapse with *n* equally-sized fingers. The relative cleft potential can be calculated using equation B1.5 for the radially-symmetric synapse (v_{sym}) and equation B4.4 for the finger-like synapse (v_{fin}), while assuming that the cleft potential of each finger is independent from each other:

$$\frac{v_{fin}}{v_{sym}} = \frac{{r_{fin}}^2 R_{ex} C_m v'_{pre}(1 - x^2/r_{fin}^2)}{2h} \frac{4h}{r_{sym}^2 R_{ex} C_m v'_{pre}(1 - x^2/r_{sym}^2)}$$

$$= \frac{2r_{fin}^2(1 - x^2/r_{fin}^2)}{r_{sym}^2(1 - x^2/r_{sym}^2)} \qquad \text{(B5.1)}$$

This gives at the center (x=0) a ratio of:

$$\frac{v_{fin}}{v_{sym}} = 2\left(r_{fin}/r_{sym}\right)^2 \qquad \text{(B5.2)}$$

To compare the VC prespike of a radially-symmetric synapse and a fenestrated synapse, we assume that the currents from each finger are synchronized and that they sum linearly. We use equation B2.2 and equation B4.5:

$$\frac{n\, i_{fin}}{i_{sym}} = \frac{2nR_{ex}C_m^2 v''_{pre} r_{fin}^3 L}{3h} \frac{8h}{\pi R_{ex} C_m^2 v''_{pre} r_{sym}^4} = \frac{16nLr_{fin}^3}{3\pi r_{sym}^4} \qquad \text{(B5.3)}$$

We assume that the total contact surface is conserved, so the surface of the fenestrated synapse ($n\ 2r_{fin}\ L$) and the radially-symmetric synapse (πr_{sym}^2) should be equal. Therefore $L = \pi r_{sym}^2 (n\ 2r_{fin})^{-1}$, which we insert into equation B5.3:

$$\frac{n\, i_{fin}}{i_{sym}} = \frac{16nr_{fin}^3\ \pi r_{sym}^2}{3\pi r_{sym}^4\ n\ 2r_{fin}} = \frac{8}{3}\left(r_{fin}/r_{sym}\right)^2 \qquad \text{(B5.4)}$$
