# Supplementary figures and images for: Using ephaptic coupling to estimate the synaptic cleft resistivity of the calyx of Held synapse
Source: PLoS Comput Biol. 2021 Oct 26;17(10):e1009527. doi: 10.1371/journal.pcbi.1009527 (PMC8570497; doi:10.1371/journal.pcbi.1009527)

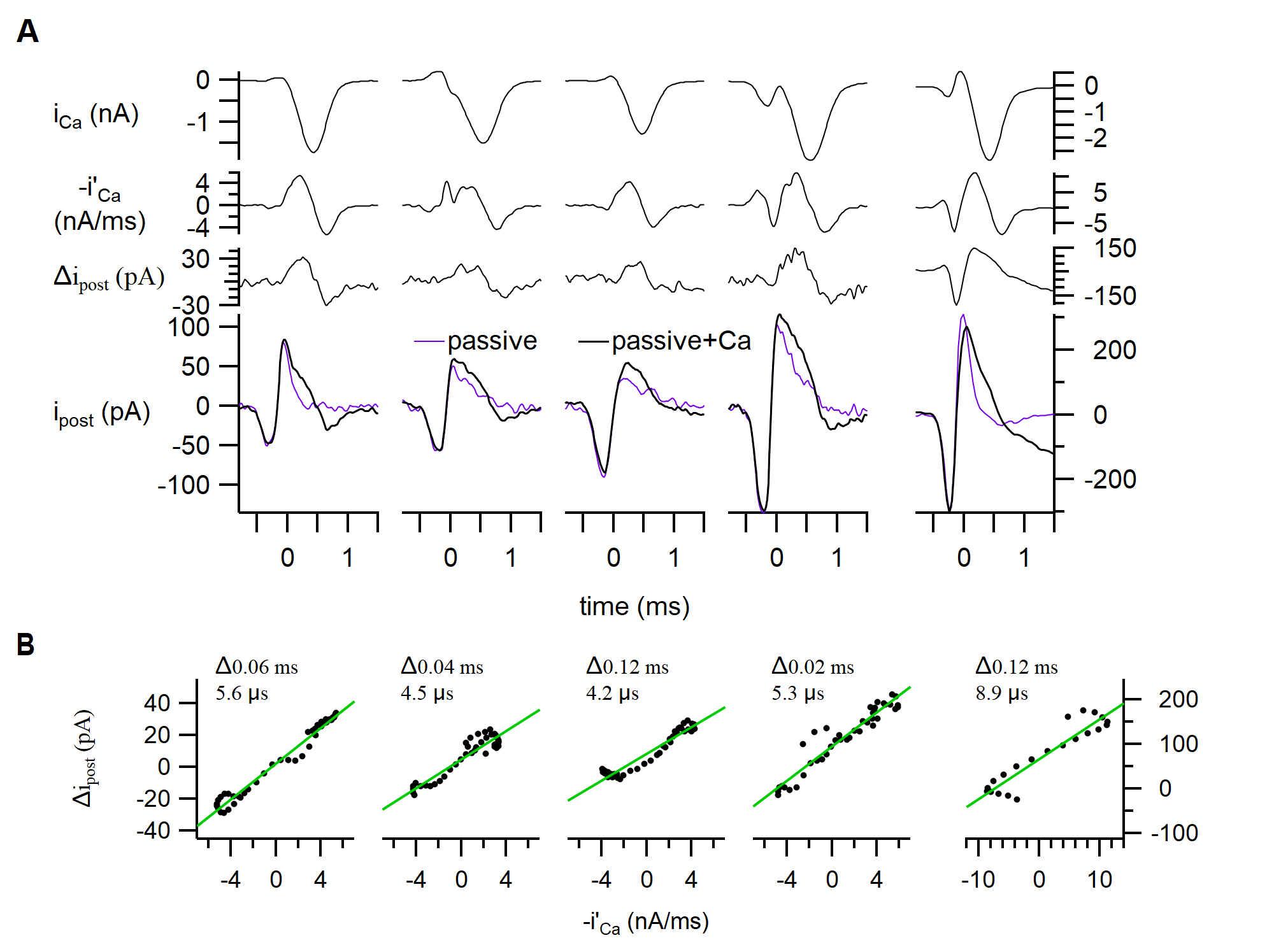

Supplement: S1 Fig — Our data set was composed of five double recordings. The presynaptic voltage-clamp waveform is shown in Fig 4A. (A) from top to bottom: The presynaptic calcium current (after P/5 subtraction), its inverted first derivative, the calcium prespike, and the active (black, thick) and passive prespike (magenta, P/5-scaled). Voltage-gated sodium and potassium currents were blocked (see Materials and Methods). The left-most column is also shown in Fig 4A. The right-most is the only one plotted on scaling of the axes on the right. Notice how even when the presynaptic calcium current does not smoothly follow a Gaussian function, its first derivative resembles the calcium prespike as predicted from our analytical relations for the resistive dissipation scenario. (B) Relation between the inverted first derivative of the presynaptic calcium current and the calcium prespike (black dots). Maximum correlation was obtained by introducing a delay for the first derivative. This delay and the slope of the regression line are shown in the top left corner of each panel. Each panel corresponds the example in the same column in A. (TIF) [file pcbi.1009527.s003.tif]
