# Supplementary material for: Using ephaptic coupling to estimate the synaptic cleft resistivity of the calyx of Held synapse
Source: PLoS Comput Biol. 2021 Oct 26;17(10):e1009527. doi: 10.1371/journal.pcbi.1009527 (PMC8570497; doi:10.1371/journal.pcbi.1009527)
Supplement: Attachment — Submitted filename: Sierksma ephaptic coupling PLOS rebuttal 2021.pdf [file pcbi.1009527.s004.pdf]

Rebuttal for PLOS Computational Biology (PCOMPBIOL-D-21-00785) "Using ephaptic coupling to estimate the synaptic cleft resistivity of the calyx of Held synapse".

We want to thank the three expert reviewers for their instructive and critical comments, but also for their positive feedback on our manuscript, which included that our study had 'high stand-alone quality'(Reviewer 1), and that it was 'original, carefully constructed and interesting'(Reviewer 3) with 'thorough and rigorous analysis with high level of detail' (Reviewer 2). Based on their comments, we have rewritten our analytical derivations without phasors to make it more comprehensible how each current (resistive, capacitive, voltage-gated) contributes to the cleft potential and prespikes. We believe that we have been able to address the different, non-overlapping issues raised by each of the three reviewers, as detailed below. We would therefore like to resubmit our manuscript and hope that the revised manuscript will be considered suitable for publication in PLOS Computational Biology.

Reviewer #1: In the manuscript by Sierksma and Borst, the authors addressed a phenomenon of ephatic coupling between the pre- and postsynaptic neurons that occurs due to a capacitive transmission of the voltage transients between cells and due to a finite conductance of the synaptic cleft dissipating the spatially distributed currents generated by the opposing membranes. This effect is most prominent at the synapses with large surface area and large currents, therefore a specialized auditory synapse, the calyx of Held, at studying which the author's laboratory has renowned expertise, was used as a model system to validate and further explore the theoretical outcomes.

Using a phasor-based analysis approach to solve the equivalent circuit of the synapse that incorporated cleft resistance and cleft capacitance, the authors derived a compact analytical solution for postsynaptic prespike well measurable at MNTB principal cells in either current-clamp or voltage-clamp modes. The derived solution predicted that a "passive" component of the prespike current recorded in voltage-clamp should be proportional to the inverted second derivative of the presynaptic AP transient, and an "active" contribution from the presynaptic calcium current – proportional to the inverted first derivative of that current. The authors used recordings from their previously published datasets to validate the predictions of phasor analysis, which worked out well, and further used the analysis to estimate the cleft conductance. Next, numerical simulations were used to model a possible contribution of sodium and potassium voltage-gated currents to the prespike, however due to a mismatch with the experimental data it was concluded that sodium and potassium channels were largely excluded from the synaptic cleft. Finally, using numerical simulations, the authors demonstrated that accelerated "mature" AP waveform would have resulted in increased cleft potential leading to suppression of AP-driven gating of the presynaptic Ca current, if the synapse geometry did not change during maturation. Developmental fenestration of the presynaptic terminal was proposed as a natural mechanism to reduce the cleft potential, and quantitative estimates of developmental changes in the cleft parameters were made accordingly.

The manuscript is clearly written, theoretical derivations appear correct, and were scholarly described in the Appendix. The experimental data, even though revisited from previous publications, are of high quality and no further experiments are required within the scope of the

study. The conclusions are well justified and discussed. However, despite a high stand-alone quality of the study, there are certain concerns regarding the conceptual novelty and the novelty of some conclusions (see major points below). Given these points can be addressed, it could render the manuscript suitable for publication after a round of revision that is not expected to take long.

Major points:

- As the authors well acknowledged in Introduction, the phenomenon of ephatic coupling has been previously addressed by a number of studies. Here, a novel way was devised to solve the circuit (Figure 2B) using phasors. However, as the authors also admitted, a compact analytical solution with phasors is only possible under certain simplifications (Y1 and Y4<>4*pi*f*Ccl; see Appendix) which effectively reduced the model circuit to a single-compartment configuration. While in reality, the proximal axonal heminode containing sodium channels (Leão et al 2005 J Neurosci, not cited) connected to a “passive” terminal with extrasynaptic and cleft-opposing membranes facing different transmembrane voltages, together assume at least two (or rather three) functional compartments (see Savtchenko 2007, ref. 30; also apparent from the results in this manuscript, Figure 5). On the one hand, the phasor predictions result in elegant expressions and agreed well with experimental data, but on the other hand this solution is valid only for particular cases like capacitively transmitted “passive” prespike and the “active” contribution of symmetric calcium current. For other, very important and interesting questions (Figures 5, 6) the authors had to switch to numerical simulations solving the system of coupled differential equations as it was done previously (e.g. ref. 30). The authors should better elaborate on the advantage of introducing phasors to this problem as compared with the approaches used previously, and state whether it was/was not possible to infer the electrical properties of the synaptic cleft using previously existing solutions.

Reviewer 1 raises three issues here: (1) is the compartmental model too simplified? (2) are phasors useful for this problem as they cannot capture the detailed behavior of voltage-gated ion channels? (3) were previously-existing solutions sufficient to infer the electrical properties of the synaptic cleft? We will answer them in turn.

(1) is the compartmental model too simplified? The purpose of our model was to infer electrical properties of the synaptic cleft using the coupling between the pre- and postsynaptic compartment underlying the prespike, and we prefer the simplest model that can do this successfully. Obviously, this model falls short in explaining detailed gating of Na- or K-currents, or other detailed aspects associated with synaptic transmission, such as the propagation of the action potential along the axon or the invasion of the action potential into the terminal, but these questions were outside our scope, and have already been addressed by Savtchenko 2007 (ref. 30) and e.g. Spirou et al. 2008 (ref. 57). For the analytical derivations we needed additional simplifications, as pointed out by the reviewer, but we believe that these simplifications were not the subject of the comment of Reviewer 1. We believe that we can safely neglect postsynaptic contributions other than what is already discussed in the Ms. because of the small size of the prespike-related currents or voltages in the postsynaptic cell. However, we did not discuss in the Ms. whether additional presynaptic compartments are likely to make a considerable contribution to the prespike. Most notably, the axon connected to the calyx also generates a capacitive current. However,

this current is: (a) smaller than the current generated at the calyceal, cleft-facing membrane (3 pF times 250 V/s gives 0.75 nA versus ~3 nA in the cleft), (b) much further away from the postsynaptic cell than the cleft currents (the path around the calyx to the postsynaptic cell would be 3-10 µm, instead of 30 nm via the synaptic cleft), and (c) the dissipation of the current will be largely uniform in the interstitial fluid (instead of restricted by the cleft-facing membranes). The contribution to the extracellular field potential thus becomes submillivolt (Holt & Koch 1999, doi: 10.1023/A:1008832702585), while we calculate the cleft potential to be about 3-10 mV. We therefore conclude that the contribution of an additional axon compartment to the prespike can be neglected. We added a brief discussion of this point to the Discussion at line 524: 'This extracellular field potential outside the cleft cannot generate a 0.1 nA-prespike amplitude intracellularly without assuming an obviously non-physiological postsynaptic admittance (~0.2 µS). Incidentally, this also indicates that other compartments, such as the presynaptic heminode, contribute very little to the generation of the prespike.'**.**

(2) are phasors useful for this problem as they cannot capture the detailed behavior of voltage-gated ion channels? One major advantage of using phasors, and of working in the frequency domain, is that, instead of solving differential equations in time, analytic solutions can be obtained by multiplication/division. As it is only an analytical tool which is not often used within the field, we decided to rewrite everything without phasors. We also think this makes the exact contribution of other currents, besides the passive currents, easier to grasp as pointed out by Reviewer 1. These coupled differential equations were defined slightly differently in ref 30, but analyzed very differently from what we do in this Ms. We have added the reference in the methods and the Appendix. We obtained the same solutions. We have now also generalized the equations showing the relation between any presynaptic cleft current and the prespike, instead of the special case of a current with a Gaussian time course.

(3) were previously-existing solutions sufficient to infer the electrical properties of the synaptic cleft? While others already indicated that the prespike likely derives from capacitive currents (Borst et al. 1995 ref 36, Von Gersdorff and Borst 2002 ref. 35, Savtchenko 2007 ref 30), none of them gives a quantitative prediction of the prespike waveform. In fact, (to our knowledge) the only discussion of the shape of the prespike was in Borst et al. (1995, ref 36), who states it to be bi- or triphasic, and that it "resembled an inverted and scaled version of the first derivative of the presynaptic action potential". With our analysis we show that the prespike could resemble either the inverted first or second derivative, but does actually resemble the scaled, inverted second derivative of the presynaptic AP. Without a clear idea of how the prespike is generated, any previously-existing solutions cannot be easily used to infer the electrical properties of the synaptic cleft.

- Based on the computed prespike waveform (Figure 5), the authors noted that homogeneous distribution of Na-channels (VGSCs) in the calyx of Held terminal was not compatible with the experimental observations and thus concluded that VGSCs are excluded from the cleft opposing membrane. Furthermore, the prespike analysis was proposed as a method to analyze the presence of different conductances at a synapse (lines 310-314). However, the authors did not cite a study (Leão et al 2005 J Neurosci) demonstrating the absence (at least a low expression level) of VGSCs at the terminal, which should be introduced to support the computational outcome. In

addition, the authors could extrapolate to the situation when VGSCs are present (e.g. at hippocampal mossy fiber boutons, Engel and Jonas 2005, Neuron; not cited) and possibly provide a reference to a study that documented a prespike waveform with a sharp spikelet contribution from Ina at such synapses (if such evidence is available in the literature).

We thank the Reviewer for pointing out our omission, and adjusted the text to include the reference to Leao et al. 2005 as well as Berret et al. 2016 and Kim et al. 2016. (Line 288: 'However, consistent with previous observations that the terminal is mostly devoid of sodium channels [47-49], these fast changes were not observed in the prespike. We therefore conclude that there is no substantial contribution of sodium channels at the release face to the prespike'**).**

We have found only a few studies reporting on pre/postspikes at other synapses (vestibular calyx: Contini et al. 2017, chick ciliary calyx: Martin & Pilar 1963, 1964, Hess and Pilar 1969; LGN Eysel, 1976), but a contribution of sodium channels was not demonstrated, and the exact relation between the action potential and the ephaptic current was not investigated in these papers. It is also important to keep in mind that the presence (or absence) of I_{Na} in an outside-out patch does not demonstrate the presence (or absence) of sodium channels in the cleft-facing membrane. Outside-out patches of the axon initial segment, a stretch of membrane known to be densely populated with sodium channels, do not contain a higher density of sodium channels than at the soma (Colbert & Johnston 1996 (doi: 10.1523/JNEUROSCI.16-21-06676.1996), Colbert & Pan 2002 (10.1038/nn0602-857), Kole et al. 2008 (doi: 10.1038/nn2040)), possibly due to anchoring of the sodium channels to the cytoskeleton which pulls them out of the outside-out patch. Please also note that Leao et al. 2005 did find I_{na} in some outside-out patches, similar to Engel and Jonas 2005. Our findings provide independent evidence for the lack of sodium channels in the calyceal terminal, which is still not universally accepted. Our approach may also be used at other synapses to determine whether or not specific ion channels are present at the synaptic release face.

We have not found a paper that found a contribution of i_{Na} in prespike recordings of other synapses (vestibular calyx, auditory inner hair cell, endbulb of Held, cerebellar pinceau, cerebellar mossy fiber-granule, cerebellar mossy fiber-unipolar brush, Purkinje terminals, chick ciliary calyx, hippocampal mossy fiber-CA3).

- The authors claimed (lines 75-77) that the prespike "is probably due to ephaptic coupling of the presynaptic AP via the synaptic cleft [30, 35], but the precise relation between the presynaptic AP and the prespike has not been studied". However, the study by Savtchenko 2007 (ref. 30) clearly showed the waveform of a capacitive prespike (Figure 3A therein) and its dependence on synapse radius and resistivity. Moreover, the same study (ref. 30) has discussed the effect of developmental fenestration of the calyx of Held synapse on the reduced ephatic coupling due to reduction of the cleft potential, with geometrical considerations very similar to those made in the last part of the manuscript. Thus, it seems necessary to provide more credit to previous studies (in particular, ref. 30) when modelling the effect of developmental fenestration on the cleft potential (pages 19-22).

Reviewer 1 indicates two points where we should have provided more credit to previous work, in particular reference 30: (1) the dependence of the prespike on the presynaptic AP, synapse radius and resistivity, and (2) the effect of developmental fenestration on the cleft potential. With regard to the first point, we have added that "larger synapses have larger prespikes [30]"at line 352, and added references at line 355. With regard to the second point, we have added the sentence (line 383) "The relation between fenestration and the coupling between pre- and postsynaptic membranes has been studied in numerical simulations [30]."

Minor:

- Figure 1 legend, line 96 ("Note the small upward deflection…"), and line 309 ("upward inflection in the CC prespike and such an inflection can sometimes be observed"): for clarity, please mark these inflections with arrows on the Figures 1D and 5B.
We have added arrow heads to indicate the putative calcium components.

- Please add an annotation "ica" in the Figure panel 3F, for compatibility with other panels. **– done.**
- Line 179: "Figure 2F" should read "Figure 3F". **– done.**
- Line 221: "we averaged the postsynaptic responses to the P/5 AP waveform" – please indicate the amplitude of unitary responses to P/5 AP waveforms (was it feasible to measure?). Did it correspond to a prediction made by A2.6, i.e. few tens of pA?

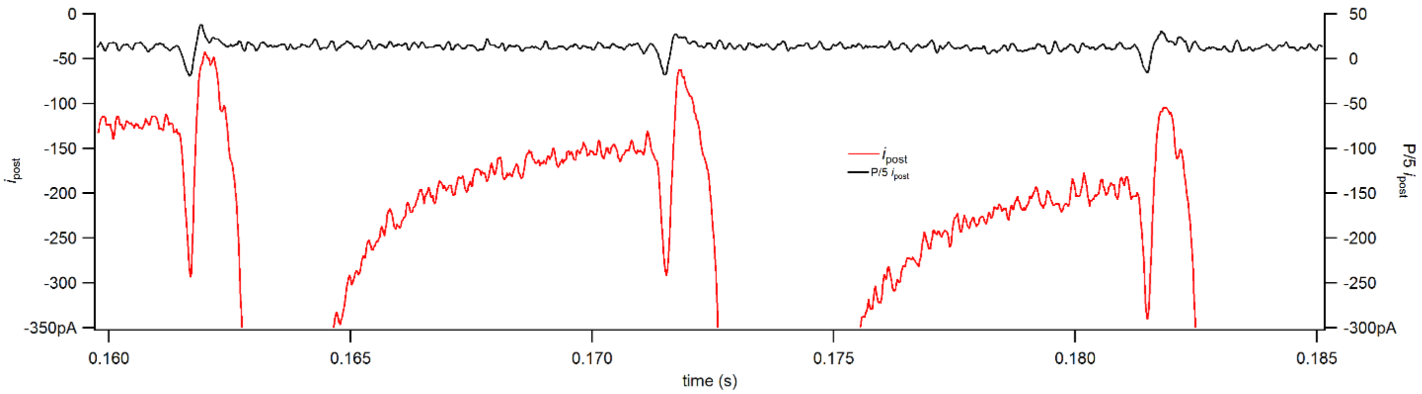

In figure 4 and S1 you will find examples of the scaled version of P/5 prespike, and above you see another example (black) together with the prespikes that are generated with the full-scale waveform. The traces were vertically shifted to improve their visibility. The amplitude of the P/5 waveform is not sufficient to open the calcium channels, while the full-scale waveform does open the calcium channels. This is also observed postsynaptically, as the prespike is much larger with the full-scale waveform, and its shape now includes the calcium prespike. Following the presynaptic calcium influx, presynaptic release of the glutamate leads to the large EPSC that follows the prespike (see also Figure 3). The P/5 prespike is indeed in the order of tens of pA. We have also added the description of the P/5 subtraction and the averaging procedure to the Methods section (line 612): 'The presynaptic passive (capacitive) component was estimated by presenting the same train five times at 5x

smaller amplitude, and summing the responses (P/5 method). The capacitive component of the prespike was isolated analogously from the same reduced APW train.'

- Figures 4 and 6: please choose better contrasting pairs of colors (blue vs black are very hard to discriminate even on a screen) – **We have improved the contrast of the graphs by combing both color-thickness or color-marker type differences to make the discriminations easier.**

- In the appendix A, please briefly introduce the meaning of atan2() function for a broader readership. **– no longer applicable**
- Lines 938 (A1.5) and 939: for consistency, please use notation "gcl" instead of "g3" **– n/a.**
- Line 970: a closing bracket is missing in denominator **– n/a.**
- Line 604: in Methods, please explicitly specify the default parameters of IntegrateODE solver (Runge-Kutta which order, integration step selection) – these parameters do matter for integration over rapidly changing transients like Na-current **– done.**
- Unformatted references found on lines 49, 63, 400, 401, 578, 579, 580, 591 **– done.**

Reviewer #2: In this study, Sierksma and Borst take an effort to quantitatively describe the ephatic coupling between pre- and postsynaptic compartments of the calyx of Held. The authors derive a theoretical framework to predict postsynaptic membrane potential changes and postsynaptic currents caused by ephatic coupling of presynaptic action potentials that precede the synaptic, neurotransmitter-mediated response (prespike). The theory is sound and relies on the assumption of a leak conductance along the synaptic cleft and a sequential arrangement of capacitors towards the postsynaptic cell. The equations derived in this study are used to simulate postsynaptic responses which are contrasted to previously published experimental data. The authors propose a relevance for presynaptic Ca2+ currents for the shape of the signals. The predictions indeed bear resemblance of experimental recordings and the authors can estimate the extracellular cleft resistivity. The authors furthermore consider how geometrical properties impact this and the prespike.
This is a thorough and rigorous analysis with high level of detail regarding the theoretical derivation of equations to calculate responses from equivalent circuits. Though the study focusses on a re-analysis of previous experimental data, the developed theory and analysis are novel in the sense that they can provide a framework to explain prespikes that have been observed at Calyx of Held type synapses under certain conditions.

We want to thank Reviewer for the kind words regarding our study.

However, though the example traces show agreement, the study falls short in providing a systematic and quantitative comparison of the predicted responses with the experiment, making it difficult to assess how well the theory overall describes the observation and how robust the phenomenon itself is (i.e. how stable the parameter values are).

This point is addressed below (Major point 1).

The observation of prespikes is limited to few specialized synapse types with large surface area (such as the immature Calyx of Held described here) and it does not become clear whether and how the conclusions of this study can be generalized to other (i.e. the majority of) synapses and what the functional relevance of the prespikes may be. The authors provide some interesting hypotheses of putative functional relevance at the calyx in their discussion, but the study would benefit greatly if these were developed further and evaluated in the context of the determined parameters towards direct predictions of the impact on synaptic function under physiological conditions. Thus, while the study will surely be of interest to specialists, it currently does not address a broader audience.

We have studied the calyx of Held synapse because of its accessibility for detailed biophysical measurements. The measurements used here for the derivation of the cleft conductance are not easily repeated in small synapses. Whereas we realize that the Ms. would appeal to a broader audience if we would show general applicability, in general we are hesitant to make strong predictions about other synapses as the current literature contains highly variable estimates for both the cleft conductance (1.4 nS to 40 µS) and cleft resistivity (50-500 Ω cm). We therefore hope that -as has happened in the past- measurements done for the first time at the calyx of Held synapse will be followed by measurements at more conventionally sized synapses; for this the methods developed in our

Ms should be helpful. These measurements would thus allow to address the generalizability of our findings more forcefully. In the meantime, we discuss the significance of the prespike in detail in the Discussion in the section "The prespike: what is it good for?", the possible differences with estimates of cleft conductance in the section "Comparison of cleft electrical properties across synapses" and the possible relevance of fenestration for other synapses in the section "Divide and conquer". We discussed at length to what extent the assumptions used for our analytic equations are met at other synapses. Prime examples where this should be possible are our unexpected finding that the cleft conductance for a radially symmetric synapse is independent of its radius (Appendix B1), and the simple relationship between extracellular resistivity and cleft conductance (Appendix B3).

Major

1. A more quantitative evaluation of the theory is needed. In the section entitled "Experimental validation of the model" the authors show comparisons of their theoretical prediction of the prespike compared to experimental measurements. The authors state that "The prespike generally matched the scaled inverted second derivative of the AP", but only one example trace is shown here and the overall congruence between the traces in panel A is not too striking (by optical inspection). The authors provide some more quantitative comparison in panel B, which is good, but the measure (peak-peak) has rather limited capacity to describe the overall shape of responses as it heavily relies on the presence/detection of the negative-going peak. Here it would be necessary to see more examples and e.g. fit the parameters for each trace by minimizing the deviation between simulation and experimental traces (using e.g. a Chi^2 value). Is optimal overlap achieved for a unique set of parameters? A comparison of Chi2 values for the two models (scaled/shifted) AP' and AP'' might provide an even clearer picture as to how superior the proposed theory is. Moreover, it would be informative to see a systematic quantification of the best fit parameters/scaling factors/phase shift for all recordings.

We have rewritten Appendix A1 to put more emphasis on the distinction between the two scenarios in which the prespike either resembles the (inverted, scaled) first or the second derivative of the presynaptic AP. This hopefully makes it more evident that the much better description by the second derivative of the peak-to-peak delay (Figure 3) does provide strong evidence in favor of our model.
Moreover, in our revised Ms., we have added additional analyses to test a second prediction of our model. We used trains in which presynaptic APs that were elicited by afferent stimulation were recorded in current clamp, while at the same time the postsynaptic currents were recorded in voltage clamp. In these 13 paired recordings, it was possible to study the relation between the waveforms of the presynaptic APs and of the prespikes during the train. Our model predicts that the relation between the amplitudes of the prespike and the amplitudes in the second derivative should be near-linear and cross the origin for the resistive dissipation scenario. If, however, the prespike originates from a high-resistance cleft, then this should hold for the first derivative. For <u>all</u> 13 train-recordings, the second-derivative yielded a better linear correlation than the first derivative (AP' $r = -0.967 \pm 0.018$ vs. AP''$r = -0.979 \pm 0.017$, paired t-test, $p = 0.00017$). Secondly, we quantified the deviation from the origin as the absolute value at 0 V/s or 0 V/s^2. In agreement with the resistive dissipation scenario, the second-derivative crossed closer to the origin than the first-derivative (AP' deviation = 122 ± 145 pA vs. AP'' deviation = 20 ± 37 pA, paired t-test, $p = 0.0060$). These new analyses are now part of Figure 3 of the Ms.

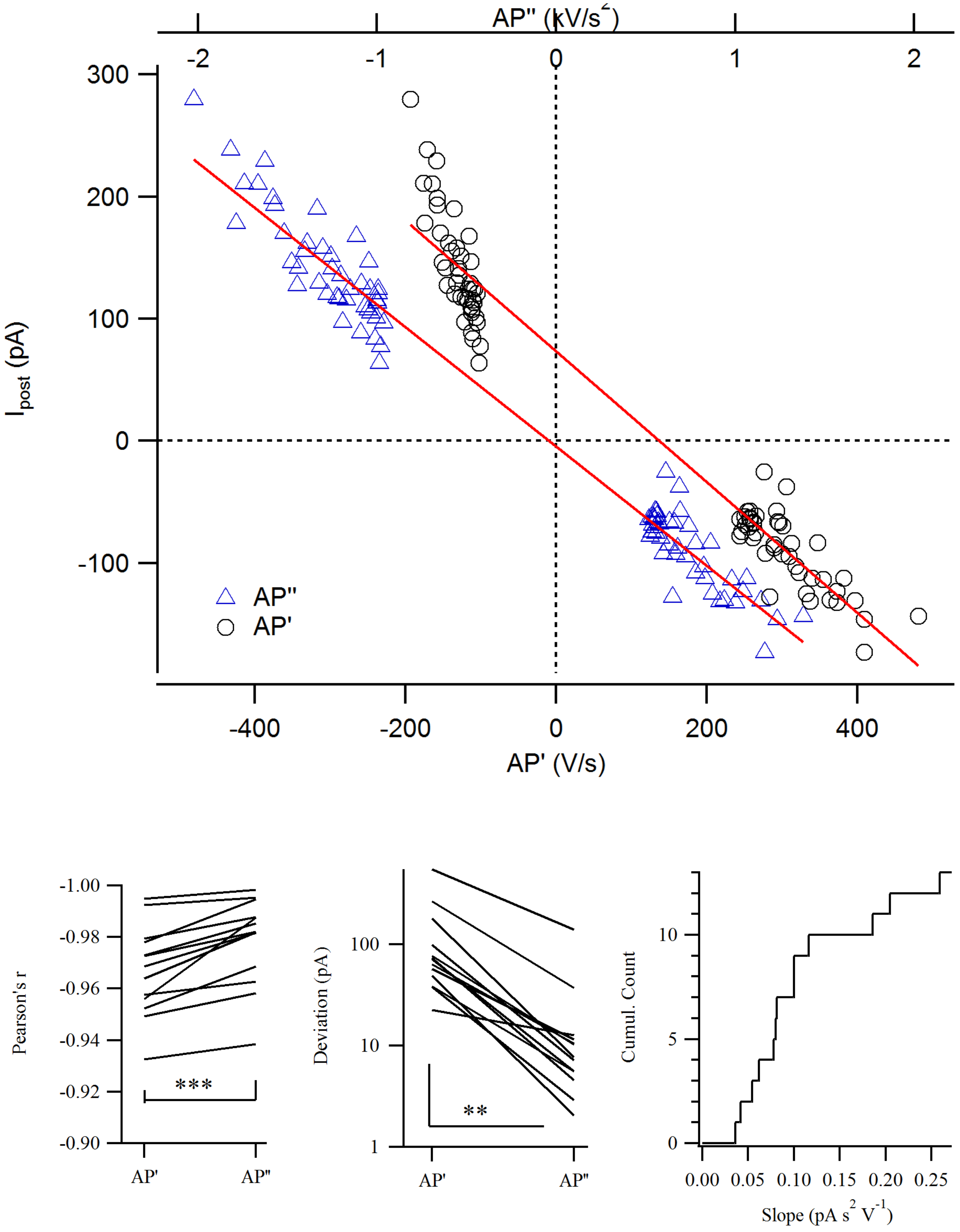

We have adjusted Figure 4 to show both the inverted second derivative corresponding to the passive prespike, with the calcium current and its inverted first derivative. This clearly

shows how well the prespike is described by the inverted second derivative of the presynaptic AP + the inverted first derivative of the calcium current.

Lastly, we now included supplementary figure S1 in Appendix A to show all five calcium prespike recordings in further support of our findings. For every recording the calcium prespike clearly resembled the inverted first derivative much better than the inverted calcium current.

2. The study would benefit if its descriptive nature could be extended to use the theory to make a specific, quantitative prediction that could either be tested experimentally in the future or be used to investigate novel aspects in existing datasets. Some suggestions might be (but are not limited to) the following: One interesting aspect is the simulation of AP-induced presynaptic Ca2+ currents and their dependence on the cleft's resistivity, but this remains rather preliminary. Which type of physiological stimuli might be impacted and how? The study following brevican deletion cited in the discussion appears to back up the author's prediction, but is there a more quantitative argument that can be made (e.g. taking into account the change in cleft width)? Maybe the authors' theory can be used to make some predictions regarding the bi-directional nature of the ephatic coupling (using the estimated parameters to predict a putative presynaptic effect by postsynaptic receptor activation)?

We appreciate the efforts of the Reviewer to consider the potential impact of our findings. In Figure 6 we indeed studied the impact of changes in the cleft potential on the gating of the presynaptic Ca channels. This is therefore an effect of presynaptic capacitive currents on presynaptic Ca currents. The impact of changes in the cleft potential that are due to postsynaptic currents via ligand-gated ion channels has received comparatively much more attention in the literature. This was for example already studied in detail for the NMJ by Eccles and Jaeger (1958, ref. 7). For the calyx, the impact of the postsynaptic currents on the presynaptic AP was already studied in detail using simulations in ref. 30, which is why we did not revisit this topic in our Ms.
We agree with the reviewer that the brevican KO is potentially very interesting, but a full study of the changes in morphology and electrotonic properties of its calyx of Held synapses lies well outside of the scope of our paper.

Minor
1. Indicate (e.g.) by arrows in Fig. 1A, B where the signals are measured that are shown in Fig. 1C. **– done.**

2. It is stated that one assumption is that the capacitance facing the cleft is equal to the postsynaptic one. This rationale should be explained further.

The calyx (axon terminal) covers a large part of the target soma. As its curvature is therefore similar to that of the postsynaptic cell, the presynaptic cleft-facing capacitance will be near-equal to the postsynaptic cleft-facing capacitance. This may not always hold true for small synapses. For instance, if a spine with a diameter of 100 nm partially penetrates an axonal bouton, the postsynaptic cleft-facing surface may be similar to a half-sphere ($0.5\pi d^2 = 0.13\ \mu m^2$), while the axonal bouton may then have a larger diameter due to

the cleft (say 100 nm + 2*25 nm = 150 nm) and therefore a larger cleft-facing area (0.28 µm^2). The capacitance scales with the area. We have added a sentence at line 146: ‘Due to its giant size, the presynaptic and postsynaptic capacitance facing the cleft in the calyx of Held synapse are close to equal (c_{syn}=c_{pre}=c_{cl}) [41, 42].’

3. Can the authors explain how non-perfect postsynaptic capacitance compensation in the VC experiment might affect the estimation?

Because of the large amount of series resistance compensation employed in the postsynaptic voltage clamp experiments (typically >90%), the capacitance compensation has to be quite precise, else oscillations are inevitable and, in particular, a strong ringing from the stimulation would be observed. Such ringing would lead to a significant overestimation of the VC prespike. Larger prespikes would then affect the estimates by underestimating the cleft conductance and overestimating the cleft capacitance. Undercompensation of the capacitance would slightly filter the recording, which would make all the very fast responses appear smaller, and would have the opposite effect on the estimation of the cleft conductance and the cleft capacitance. Capacitance compensation may have a large impact, but we consider this to be unlikely in our experiments.

4. Please indicate how Ca2+ currents were blocked in the experiment depicted in Fig. 3 directly in the figure legend to make it easier for the reader to obtain this information.

These recordings did not involve calcium blockers. To record the presynaptic calcium current properly, one needs to subtract the passive currents, related to the voltage-clamp waveform, from the actual recording. This is done by first repeatedly playing a smaller waveform (P/5, i.e. 1/5 of the amplitude of the full waveform) which generates the passive currents, but is not sufficiently large to open the calcium channels, followed by the full-scale waveform, which generates not only the passive currents but also the calcium currents. During the P/5 protocol small prespikes can already be observed (see below, traces were vertically shifted to improve visibility). These prespikes were averaged and scaled to be 5x larger, which we call the passive prespike as it corresponds to the passive currents of the cleft-facing membrane. The difference between the passive prespike and the prespike generated by the full-scale waveform corresponds to the calcium prespike. We have added a description of this procedure to the Methods section.

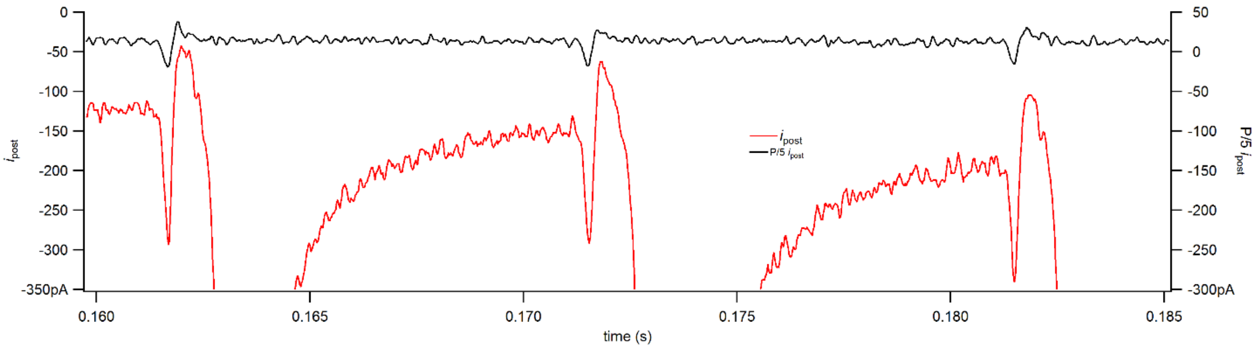

Reviewer #3: This MS analyses the effects of the presynaptic action potential at the calyx of Held on the voltage in the synaptic cleft by exploiting postsynaptic recordings, in which, under some conditions at least, a signal reflecting the presynaptic action potential can be observed. The authors construct a plausible equivalant circuit of the calyx, enabling them to identify the fundamental mechanisms governing these processes. In particular, they conclude that the peak voltage in the cleft scales with the 4th power of its radius. In addition, the authors show that the presynaptic calcium current can also be investigated via its effect on the cleft potential. The authors are also able to account for the effects of the morphological changes occurring during development.

Overall, I found this paper to be original, carefully constructed and interesting. In my opinion the modelling is quite judicious, with an informative level of approximation and a thorough exploitation of available data. I particularly enjoyed the ability to analyse the calcium current.

I have one major concern, which is that in trying to check the equations of the modelling, I fell at the first fence. I was unable to verify equation A1.1 (in the Appendix). Specifically, in my attempt I obtain

V_cleft/V_pre = jY_2/(Y_3 + j(Y_2 + Y_5))

i.e., without the minus signs. I don't believe the two versions to be equivalent. Maybe the authors are using a non-standard version of complex impedance?

Obviously, I would like the authors to check this equation and, if there is indeed an error, investigate how it affects the rest of the modelling/analysis. I haven't attempted to calculate how great an effect the apparent error would have, but the equation is quite fundamental to the remainder of the paper. The authors may find it beneficial to employ explicit circuit modelling software (such as Spice; I believe free versions are available) to provide some additional assurance that their analytical solutions are correct.

We are glad to read the appreciation of our work by Reviewer 3.
Regarding the phasor analysis, Reviewer 3 is correct that we were using a non-standard notation where, following Wavemetrics, the imaginary plane is reversed (Igor manual, Chapter III-9 – Signal Processing, page III-242, Phase Polarity, https://www.wavemetrics.net/doc/igorman/III-09%20Signal%20Processing.pdf), and where Y would indicate the magnitude of the complex vector instead of the admittance.

More generally, we realized that phasors (and complex arithmetic) are not routinely used by many of the potential readers of our Ms, and we have therefore decided to leave out the phasor analysis entirely. Instead, we derive the current relations without phasors while obtaining the same solutions.

Nonetheless, we took the advice of Reviewer 3 and checked our findings with LTSpice XVII (Analog Devices, Inc.). A screenshot is shown below. These results confirmed our findings. (V(n001) = v_{pre},, V(n002) = v_{cl}, I(C2) is the current crossing C2, V(n003) = v_{post}).

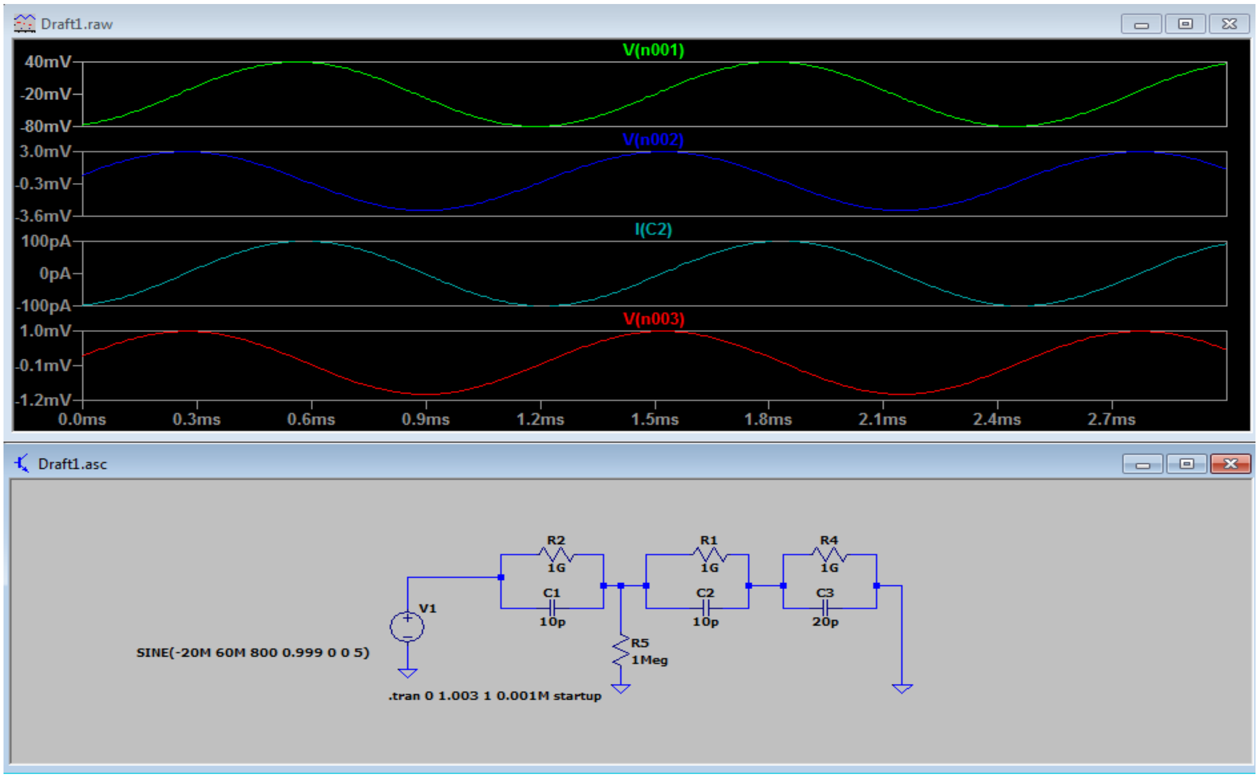

Despite this potential error, I still find the whole approach original and interesting and I look forward to discovering whether I am mistaken, whether none of the conclusions are materially affected or whether different conclusions pertain.

At this stage I have few other comments. As a matter of personal preference, I would find the figures more readable with additional labelling, indicating for instance the nature of the traces in Fig. 1A,B and Fig. 4A,C.

We again thank the Reviewer for his kind comments and we have added additional labeling to improve the clarity of Figures 1, 3, 4, 5 and 6.
